# Anaplasic lymphoma kinase positive inflammatory myofibroblastic tumour with renal pelvic calculus: a case report

**DOI:** 10.4076/1757-1626-2-6373

**Published:** 2009-08-21

**Authors:** Hinde Elfatemi, Chbani Laila, Kaoutar Znati, Mohamed Fadl Tazi, Youness Ahallal, Elmehdi Tazi, Moulay Hassan Farih, Afaf Amarti

**Affiliations:** 1 Department of Histopathology, Hassan II University Hospital InstituteFezMorocco; 2 Department of Urology, Hassan II University Hospital InstituteFezMorocco; 3 National Institute of Oncology, IbnSina University Hospital InstituteRabatMorocco

## Abstract

Inflammatory myofibroblastic tumour is a distinctive mesenchymal neoplasm, composed of a variable admixture of myofibroblastic spindle-shaped and inflammatory cells which were originally described in the lung, as a nonneoplastic lesion and designated as an inflammatory pseudotumour. The lack of certainty of the IMTs pathogenesis is reflected in the large number of terms which have been attributed to this lesion. Recent genetic and molecular studies of IMTs have showed chromosomal abnormalities of 2p23 resulting in a rearrangement of the anaplasic lymphoma kinase gene and have also provided evidence for a monoclonal, noeplastic origin for IMT.Occurrence of IMT in the kidney is very rare, and to our knowledge, only 30 such cases have been described in the literature.This report describes an original case of an ALK positive IMT of the kidney associated with renal pelvic calculus which we believe has never been reported. The differential diagnosis of IMTs will also be discussed.

## Introduction

The inflammatory myofibroblastic tumours (IMT) correspond to a distinct organic entity, made up of spindle-shaped cells myofibroblastic frays with an inflammatory infiltrate formed by lymphocytes, plasmocytes and eosinophilic cells. These lesions are ubiquitous, preferentially interesting the lung, the mésentery and the epiploon [1]. When it comes to the urogenital system, the lesions are usually located in the bladder, the renal localization being exceptional with only 30 cases reported in the literature. The IMT occurs more frequently for young patients [1]. The aetiopathogenesis of these tumors remains indistinct; however, recent molecular studies of cytogenetics and biology demonstrated the presence of genetic reorganization implying the gene ALK.

We report a new case of inflammatory myofibroblastic kidney tumour, associated with a renal pelvis lithiasis. In the meantime, we will insist on the pathogenesis discussion of these tumours and on their anatomopathologic diagnosis.

## Case presentation

A 35-year-old Moroccan man, without particular pathological history, was admitted as he was suffering from a one-year recurrent renal colic. The clinical examination of the patient revealed a big left kidney without the other associated clinical anomalies.

**Figure 1.**
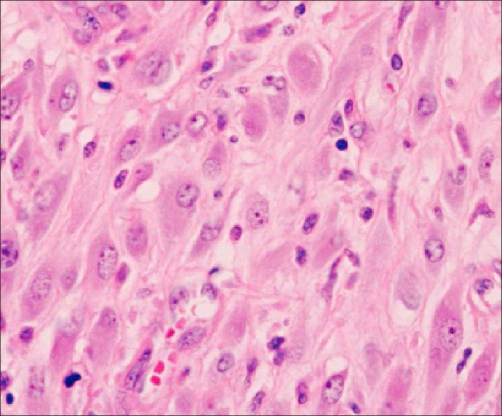
Proliferation of spindle-shaped cells (HES × 40).

**Figure 2.**
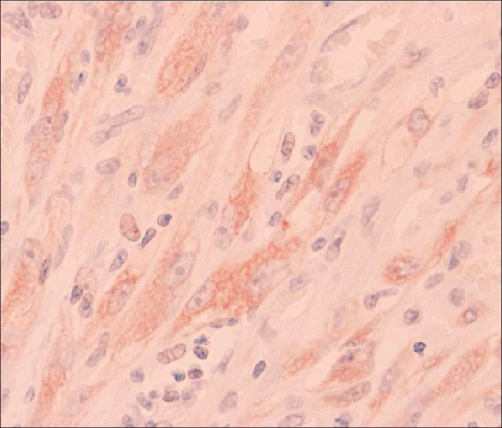
Diffuse cytoplasmic positivity with the ALK.

The biological investigation did not reveal any particular anomaly. Radiological exploration was also carried out, by the means of a renal ultrasonography and an abdominal scanner. It has unveiled a tumor measuring 5 cm of main axe, located in the lower pole of the left kidney, with an extension to the hilum. A surgery with total nephrectomy was performed on the patient in an effort to deal with this unusual renal tumour. The macroscopic examination showed a part of left total nephrectomy of 10 × 8 × 6 cm. With the opening, it contained a heterogeneous lesion of a tumoral aspect, measuring 5 × 4 × 4 cm, with blurry boundaries and occupying the lower pole of the kidney. This tumor was extended to the hilum and had no recognizable vascular structure. There was no crossing of the renal capsule. Histologically, the tumor was formed by a proliferation of spindle-shaped cells, of myofibroblastic look (Figure 1) mixed with a polymorphic inflammatory infiltrate, made by lymphoplasmocytes and polynuclear on a bottom oedématous and focal hemorragic. The spindle-shaped elements lined up in short bundles, and with atypical nuclei with rare mitosis. In periphery, the limits of the lesion were broadly defined with infiltration of fat hilum and the adjacent renal parenchyma. In immunohistochimy, the spindle-shaped cells expressed the actine smooth muscle and focally, the epithelial antigen membrane (EMA). They also showed an intense and diffuse cytoplasmic positivity (Figure 2) with regards to the ALK (Antibody antiALK-1). The immunolabelling with the antibodies anticytokératine, anti-PS100 and anti-Desmine were negative. The evolution was normal and no recurrence has been reported during a follow-up of 3 years.

## Discussion

The morphological and phenotypical features, reported in our case, are those of an inflammatory myofibroblastic tumour (IMT), characterized by spindle-shaped cells and myofibroblastic proliferation, and associated with a polymorphic inflammatory infiltration, particularly rich in plasmocytic cells which are contained within an oedematous stroma. The pathogenesis of the IMT has been for a long time in the center of a debate whether their nature is reactive or tumoral. Initially, they were described in the lung, under the post-inflammatory tumour term [2]. Later on, under a variety of anatomical websites, similar lesions were described using a range of different denominations (plasmocityc cells granuloma, pseudotumour of plasmocytic cells, inflammatory myofibroblastic proliferation, inflammatory pseudotumour), which reflects their debated pathogenesis.

Recent studies show that a part of these lesions, at the very least, constitutes a true entity of neoplasic nature. Indeed, Griffin et al. carried out a study on 3 new cases and of the retrospective analysis of the caryotypes previously published. It revealed a recurring and non-random anomaly of the chromosomal band 2p23 that is located on the distal segment of the court arm of chromosome 2 with a point of break in the ALK gene (Anaplastic Lymphoma Kinase), coding for a receiver of tyrosin kinase and already known for its implication in the anaplasic lymphoma with big cells [3]. Techniques of molecular biology, RT-PCR, confirmed the reorganization of the ALK gene and identified two other genes participating in this modification.

Therefore this analysis confirmed dismissed the reactive nature of the IMT, and confirmed they were clonal and tumoral in connection with the deterioration of the ALK gene along with the existence of various genes playing a role in this fusion.

Cases of association of IMT with some bacterial or viral infections such as HHV-8 virus (human herpes virus-8) were reported in the literature [4]. In the case of the patient introduced above, the unusual coexistence between a positive ALK IMT at the level of the kidney, with an extension to the hilum and a lithiasis of the renal pelvis allows two assumptions.

The first one is the existence of a cause-effect relationship between the tumour and the lithiasis as the tumour can support the constitution of the lithiasis by obstructing the urinary excretion. In addition, a lithiasis could be at the origin of an excessive reactive inflammatory process, carrying out a pseudotumoral aspect, however, this type of lesion gives a negative immunolabelling with the ALK, but the exact opposite is detected in our patient [5].

The second one assumption is the fortuitous association which seems more probable in the case of our patient.

The IMT of the kidney, often asymptomatic, can be revealed by lumbagos, hématuria, hydronephrosis, nonspecific systemic demonstrations such as prolonged hyperthermia or by the means of biological inflammatory syndromes [6]. Despite the advances in medical imagery, the IMT raises the preoperative problem of diagnosis with a kidney malignant tumour because of the lack of any specificity of its radiological aspect. Indeed, the IMT can be composed of a variable quantity of myofibroblasts, fibroblasts and inflammatory elements in an œdematous myxoid or collagen matrix [1]. Three morphological profiles, which can coexist within the same tumour, can therefore be individualized: a myxoid aspect, containing spangled myofibroblastic cells within a myxoid and oedématous stroma abundant in polymorphic inflammatory infiltrates, a compact aspect, marked by the proliferation of spindle-shaped cells, with provision fibrous within amyxoid and collagen heterogeneous matrix also containing inflammatory elements, lymphocytes and plasmocytes primarily. Finally, an aspect that is poor in cells, with an extensively collagenous bottom and a few inflammatory elements [1]. The immunohistochimy confirms the myofibroblastic nature of the Spindle-shaped cells proliferation with the expression of the vimentine (99%), the actine smooth muscle (92%), the desmine (69%) and the cytokeratines (36%) [1]. The expression of ALK, with a immunolabelling of mainly cytoplasmic localization in more than half the cases of the IMT with a reorganization in the genes, is an favorable argument to the diagnosis [5]. In the specific case of the patient presented above, the immunolabelling by the anti-ALK1 antibody revealed a diffuse cytoplasmic expression.

According to the morphological profile of the IMT, the differential diagnosis must include various types of tumours with spindle-shaped kidney cells, but also reactive processes. Indeed, when the myofibroblastic proliferation develops within an oedématous stroma and/or myxoi, the aspect can recall a visceral nodular fasciite. Nevertheless, the IMT contains traditionally the inflammatory infiltrates that is rich in plasmocytes, which are often missing in the nodular facciite [7]. In the case of a more compact fascicle IMT, especially when it contains atypical cells; the differential diagnosis must be done with sarcomatoid carcinoma, and particularly with a sarcoma, in specific cases of inflammatory leiomyosarcoma, inflammatory fibrosarcoma and also in cases of malignant histiocytofibroma for the IMTrich in histiocytes [8]. The ALK is not expressed in any of these lesions [4]. The lymphoma anaplasic with large cells of the sarcomatoïd type must be taken into account as well, because of the positivity of ALK when it is associated with an expression of the actine smooth muscle in particular [9].

## Conclusion

The IMT constitute a distinct tumoral entity, with a genetic reorganization of ALK with the participation of a variety of genes in the fusion and the activation of detectable ALK in immunohistochemistry. The unusual association with a lithiasis of the renal pelvis, described is probably a coincidence. The extremely rare localization of the IMT in the bladder leads to the morphological differential diagnosis with several kidney tumours with spindle-shaped cells and a visceral nodular fasciite.
